# Current understanding of epigenetics role in melanoma treatment and resistance

**DOI:** 10.1186/s12935-022-02738-0

**Published:** 2022-10-12

**Authors:** Mohsen Karami Fath, Ali Azargoonjahromi, Asma Soofi, Faezeh Almasi, Shahnaz Hosseinzadeh, Saeed Khalili, Kamran Sheikhi, Saeid Ferdousmakan, Soroor Owrangi, Minoovash Fahimi, Hamidreza Zalpoor, Mohsen Nabi Afjadi, Zahra Payandeh, Navid Pourzardosht

**Affiliations:** 1 Department of Cellular and Molecular Biology, Faculty of Biological Sciences, Kharazmi University, Tehran, Iran; 2 Shiraz University of Medical Sciences, Shiraz, Iran; 3 Department of Physical Chemistry, School of Chemistry, College of Sciences, University of Tehran, Tehran, Iran; 4 Pharmaceutical Biotechnology Lab, Department of Microbial Biotechnology, School of Biology and Center of Excellence in Phylogeny of Living Organisms, College of Science, University of Tehran, Tehran, Iran; 5 Department of Microbiology, Parasitology and Immunology, Ardabil University of Medical Sciences, Ardabil, Iran; 6 Department of Biology Sciences, Shahid Rajaee Teacher Training University, Tehran, Iran; 7 School of Medicine, Kurdistan University of Medical Sciences, Kurdistan, Iran; 8 Department of Pharmacy Practice, Nargund College of Pharmacy, Bangalore, 560085 India; 9 Student Research Committe, Fasa University of Medical Sciences, Fasa, Iran; 10 Faculty of Medicine, Comenius University, Bratislava, Slovakia; 11 Shiraz Neuroscience Research Center, Shiraz University of Medical Sciences, Shiraz, Iran; 12 Network of Immunity in Infection, Malignancy & Autoimmunity (NIIMA), Universal Scientific Education & Research Network (USERN), Tehran, Iran; 13 Department of Biochemistry, Faculty of Biological Science, Tarbiat Modares University, Tehran, Iran; 14 Department Medical Biochemistry and Biophysics, Division Medical Inflammation Research, Karolinska Institute, Stockholm, Sweden; 15 Biochemistry Department, Guilan University of Medical Sciences, Rasht, Iran

**Keywords:** Melanoma, Epigenetics, Drug resistance, Immunotherapy

## Abstract

Melanoma is the most aggressive form of skin cancer resulting from genetic mutations in melanocytes. Several factors have been considered to be involved in melanoma progression, including genetic alteration, processes of damaged DNA repair, and changes in mechanisms of cell growth and proliferation. Epigenetics is the other factor with a crucial role in melanoma development. Epigenetic changes have become novel targets for treating patients suffering from melanoma. These changes can alter the expression of microRNAs and their interaction with target genes, which involves cell growth, differentiation, or even death. Given these circumstances, we conducted the present review to discuss the melanoma risk factors and represent the current knowledge about the factors related to its etiopathogenesis. Moreover, various epigenetic pathways, which are involved in melanoma progression, treatment, and chemo-resistance, as well as employed epigenetic factors as a solution to the problems, will be discussed in detail.

## Introduction

Melanoma stems from a malignant transformation of melanocytes synthesizing melanin throughout the body as photo-protective pigments [1]. Melanoma has been rampant across the world [2], accounting for 5.5% of all new cancer cases and resulting in 7230 deaths (1.2% of all cancer deaths) just in the United States in 2019 [3]. Various factors have been considered to involve in melanoma progression [4], namely genetic alteration in multiple genes (oncogenic and tumor suppressor genes) such as cyclin-dependent kinase inhibitor 2A (CDKN2A), melanocortin receptor (MC1R), cyclin-dependent kinase 4 (CDK4), Ras, and BRAF (v-raf murine sarcoma viral oncogene homolog B1) genes. In addition, damage in DNA repair processes, changes in cell growth, and proliferation mechanisms are involved in melanoma progression. Whereas targeting epigenetic factors is deemed a novel strategy to treat melanoma patients [5, 6]. Epigenetics refers to the study of changes in gene function that are mitotically and/or meiotically heritable and that do not entail a change in DNA sequence [7, 8]. Epigenetic factors can alter the expression of microRNAs, target genes in cell growth, differentiation, or even death [9, 10].

Herein the crucial risk factors and pathways involved in the development and pathogenesis of melanoma, as well as the function of epigenetics in melanoma progression, treatment, drug resistance, and the efficiency of targeted therapy and combined immunotherapy agents, will be reviewed and elucidated.

## Pathways and different risk factors involved in melanoma occurrence and development

### MAPK and AKT signaling pathways

Genetic alterations in melanoma patients were seen to activate the RAS/RAF/MEK/ERK (MAPK) and the PI3K/PTEN/AKT (AKT) signaling pathways, so that it has been found that the growth of melanoma cells can be blocked as a result of inhibiting both ERK and PI3K signaling [11–13]. The MAPK (Mitogen-activated protein kinase) pathway can affect downstream pathways of some receptors, such as cytokine, heterotrimeric G-protein, and tyrosine kinase receptors. The small G protein Ras, which belongs to a family of hydrolase enzymes, is an anchored protein in the inner leaflet of the membrane bilayer [14–17].

So, MAPK is an essential pathway in most cases of melanoma. Several mutations, such as NRAS and BRAF can activate this pathway [18]. The most critical downstream molecules of the MAPK pathway are BRAF and CRAF serine-threonine kinases [18]. Both BRAF and CRAF were shown to have a regulatory domain (CRD), a RAS-binding domain (RBD), and a kinase domain that inhibits RAF function [18]. Contemporary, it has been reported that mutation in both NRAS and BRAF is linked with poor prognosis in stage IV of melanoma cases [18]. Such mutations are ascertained in the benign proliferation of melanocytes, metastatic melanoma, and invasive melanoma [18]. Both vemurafenib and dabrafenib, which are approved by FDA, have shown a noticeable efficacy in BRAF inhibition, thus suppressing the tumor cells [18]. Mutations of NRAS (in 15–30 percent of melanoma tumor samples) have been recognized as a vital driver of oncogenesis in melanoma patients [18]. It should be pinpointed that growth factor receptors (namely epidermal growth factor receptor, c-Kit, and c-Met) can be activated by RAS promotion in melanoma cases [18, 19].

On the other hand, it is of note that rampant genetic changes in melanoma are capable of reducing apoptosis through the overexpression of B-cell lymphoma 2 (Bcl-2), loss of both Phosphatase and tensin homolog (PTEN) and nuclear factor-κB (NF-κB), and mutation of Akt3, NRAS, and BRAF [11, 20]. Most importantly, lymphocytes and pigmented melanophages (possessing ingested melanin) are found close to the dermal-epidermal p53-positive cells, suggesting cell death among the melanocytes [21, 22]. Accordingly, after p16INK4A-dependent senescence, melanocytes can be provoked by a p53-dependent ‘back-up’ cellular senility checkpoint, thereby mediating the transformation of NRAS or BRAF [23].

### BRN2 expression

BRN2 is a member of the POU domain family of transcription factors with a crucial function in the progression and metastasis of melanoma [24, 25]. A high level of BRN2 expression could lead to elevated invasiveness as well as suppression of DNA repair and apoptosis in melanoma cell lines [26]. This fact corroborates the notion that BRN2 is involved in high somatic mutations in melanoma cases [24, 27]. Significantly, BRN2 contributes to the melanocytic-lineage oncogenic factor (MITF)-mediated progress of melanoma. MITF is the master regulator in the transcription of melanocytes [24]. An in vivo imaging study on melanoma cell lines has indicated that the BRN2 expression is increased in invasive cells of the primary tumor, while MITF expression is lost [28]. Most biopsy samples of melanoma patients and drug-sensitive melanoma cell lines have shown increased expression of MITF [29]. High expression of MITF is distinguished as a fundamental mechanism of resistance to MAPK pathway suppression [30]. Since overexpression of BRN2 and reduced expression of MITF are directly linked to activation of MAPK pathway, they are significantly associated with early resistance to targeted therapy [31]. Taken together, BRN2 is suggested as a critical regulator that is involved in drug resistance and invasion during melanoma treatment as a counterbalance to the MITF [24]. Even with well-known functions, the enormous scope for uncovering its tumor progression effects, the tumor microenvironment on BRN2, and the involved epigenetic switching mechanisms are still needed.

### Hypoxia-inducible factor-1 alpha

Hypoxia is the most commonly accruing condition among all solid tumors. It can lead to poor prognosis in cancer patients, irrespective of the kind. Hypoxia can increase the progression of tumor cells via activation of HIF-1α. This protein is responsible for regulating essential genes, which interfere in cell proliferation, angiogenesis, metabolism, and metastasis [32–36]. Hypoxia-Inducible Factor1 (HIF-1) is a transcription activator, which is sensitive to oxygen and encompasses the HIF-1β and HIF-1α subunits. HIF-1α is activated by post-translation modifications such as phosphorylation, acetylation, hydroxylation, and ubiquitination [32, 37]. HIF-1α also has a prominent role in angiogenesis by affecting cellular metabolism, tumor invasion, vascular endothelial growth factor (VEGF), cell survival, and metastasis [32, 33, 38–40]. HIF-1α expression is related to aggressive characteristics of melanoma. Hence, the incorporation of HIF-1α as a promising prognostic indicator in melanoma may add growing value to current staging procedures [41]. Accordingly, along with post-translation modifications, some signaling pathways can activate HIF-1α, including the RAS/RAF/MEK/ERK and MAPK/ERK signaling pathways [42, 43]. The MAPK pathway was shown to induce the formation of the HIF-p300/CREB-binding protein (CBP) complex and modulate the transactivation of p300/CBP [44]. The RAS/RAF/MEK/ERK pathway can be stimulated via mutations that occur in membrane receptors and oncogenic genes, namely KIT and both N-RAS and B-RAF, respectively. Hypoxia condition could also be involved in activating the JAK⁄ STAT (signal transducer and transcription activator and Janus kinases) pathway in response to cytokines and growth factors [32, 45–47]. This phenomenon can be triggered by HIF-1α in multiple cancer cell lines and animal models [47–49].

### Src and STAT3 signaling

Obliterating the STAT3 pathway is demonstrated to block oncogenesis. It has been found that STAT3 signaling disruption could lead to inducing both apoptosis and cell cycle arrest in sundry cancer cell lines. This condition occurs in the cases of prostate cancer, multiple myeloma, and breast cancer [50]. Cao et al. explored the involvement of STAT3 signaling in melanoma occurrence/development by evaluating the anti-melanoma activities of shikonin in cell and zebrafish xenograft models. It has been demonstrated that shikonin (a naphthoquinone pigment extracted from the dried root of *Zicao* (*Lithospermum erythrorhizon, Onosma paniculata, or Arnebia euchroma,* as a traditional Chinese herbal medicine) could block the phosphorylation of STAT3, decrease the levels of STAT3-targeted genes involved in melanoma survival and migration (Mcl-1, Bcl-2, MMP-2), and finally suppress melanoma growth [51, 52]. In a different study, the inhibition of the viability and proliferation of A375 and A2058 melanoma cells was shown by the dauricine via blocking the phosphorylation-mediated activation of STAT3 and Src in a dose-dependent manner [34, 53–55]. Recently, Meng et al. have also evaluated the potent anti-melanoma activity of podocarpusflavone A (PCFA). This compound has been described to inhibit melanoma growth via inhibition of the JAK2/STAT3 pathway [56]. These findings indicate that the JAK2/STAT3 pathway plays a significant role in melanoma occurrence/development.

### Ambra1 protein

Ambra1 is identified as a multifunctional scaffolding protein and pro-autophagy protein. It has been reported that functional deficiency in Ambra1 could induce hyperplasia and impaired autophagy in neuro-epithelial cells of mouse embryos [57, 58]. Ambra1 can initiate autophagy via regulation of Unc-51 like autophagy activating kinase (ULK1) and Beclin1 [57, 59]. Autophagy can lead to drug resistance and progression in multiple cancers (e.g. melanoma, acute myeloid leukemia (AML), etc.) [60–62].

Ambra1 has also been found to have a key function in cell cycle progress and proliferation by affecting the stability of c-Myc, interaction with the protein phosphatase 2A (PP2A) and stability of Cyclin D1 (CCND1) through interaction with the E3 ligase DDB1/Cullin4 [63, 64]. It was shown that concomitant loss of Ambra1 and expression of Loricrin in the peritumoral epidermis could be used as prognostic biomarkers for high-risk tumor subsets and early stages of melanoma [65, 66]. A list of onco-suppressor and oncogenic factors involved in melanoma is presented in Table 1.

**Table 1:** The onco-suppressor and oncogenic factors involved in the uncontrolled cell proliferation of melanocytes

Gene	Gene type	Function	Comment	Refs.
*MC1R*	Oncogenic	The eumelanin pigments (dark brown pigments) are synthesized in response to UV exposure by this receptor	The high expression leads to the more frequent cell division	[66]
*CDK4*	Oncogenic	Contributing to the regulation of cell cycle	Triggering metastasis inducing pathways and also, interfering the phosphorylation of pRB (retinoblastoma protein) in the mid-G1 phase	[55]
*BRAF*	Oncogenic	Contributing to regulating cell division and differentiation as a part of the family of signal transduction protein kinases	Activating the MAPK pathway involved besides RAF and the RAS family	[67]
*CCND1*	Oncogenic	In a manner dependent on cyclin-dependent kinases, or CDKs, promote progression of G1-S phase of the cell cycle by inactivating the RB protein	Contributing to the phosphorylation of pRB by binding to CDK4	[68]
*RAS* and *NRAS* (neuroblastoma *RAS* viral *oncogene* homolog)	Oncogenic	Regulating cell division by encoding N-Ras protein as GDP–GTP-regulated binary on–off switches	Activation of MAPK and the phosphatidylinositol 3-kinase (PI3K) pathway	[53, 69]
*c-KIT*	Oncogene	Interacting with stem cell factor (SCF), activating downstream signaling molecules, causing the expression of certain genes, regulating cell differentiation and proliferation, and restraining cell apoptosis, associated with tumor formation, development, migration, and recurrence	Induction of both MAPK and PI3K-AKT kinase pathways	[70]
*GNAQ* (guanine nucleotide-binding protein G(q)) and *GNA11* (guanine nucleotide-binding protein subunit α-11)	oncogene	Making a guanine nucleotide-binding protein G(q) subunit alpha (Gαq) to activate downstream cellular signaling pathways	Encoding G-protein alpha subunit q and alpha subunit 11, respectively	[71]
*P53*	tumor suppressor gene	controlling cell division and cell death in the cell’s nucleus	Associated with advanced-stage disease	[72]
*TP 53*	tumor suppressor gene	Encoding P53 protein as a tumor suppressor by keeping cells from growing and dividing	A somatic mutation causing abnormal p53 expression	[73]
*P16*	tumor suppressor gene	As a CDK inhibitor; it slows down the progression of the cell cycle	Effecting G1 cyclin-dependent kinases cell regulator	[74]
*BCORL1*	tumor suppressor gene	Encode a transcriptional corepressor binding to promotor regions of DNA binding proteins	Represseing E-cadherin expression via interaction with CtBP	[75]
*PPP2R3B* (gonosomal protein phosphatase 2 regulatory subunit B, beta)	tumor suppressor gene	As a major family of Ser/Thr phosphatase gene negatively control cells growth and division	Intervening with DNA replication and cell cycle progression by its regulatory subunit PR70	[76]
*RASA2* (RAS p21 protein activator 2)	tumor suppressor gene	Encode RasGAP as a tumor suppressor	Activation of RAS GTPase, increase RAS activation, and melanoma cell growth	[77]
*PTEN*	tumor suppressor genes	Regulate cell division by keeping cells from growing and dividing	Elimination of negative regulating on downstream components of the PI3 kinase pathway and Akt	[78]
*CDKN2A*	tumor suppressor genes	Encode the cell cycle inhibitor P16^CDKN2A^	Disruption of the function of p16^INK4a^ and p14^ARF^ effecting two cell cycle regulatory pathways, the p53 and the RB1 pathways	[79]

## Role of epigenetic in melanoma development and pathogenesis

Besides the signaling pathways and factors mentioned above, chromatin modification (through cytosine methylations), histone modification (such as acetylation, methylation, and phosphorylation leading to chromatin remodeling), and noncoding RNA (ncRNA) regulation are the epitome of epigenetic inheritance that have been identified in melanoma cases. Epigenetic mechanisms that are involved in melanoma cancer are summarized in Table 2.

**Table 2:** Epigenetic mechanisms in Melanoma cancer

Epigenetic Mechanisms	Type	Regulatory Protein	Biomarker Gene/Protein	Modification Context/Function	Expression Changes	Refs.
*Chromatin Modifications*	*Writer*	DNA methyltransferases (DNMTs)	Preferentially Expressed Antigen in Melanoma (*PRAME*)	Hypomethylation of specific CpG sites being close to the PRAME promoter leading to transcriptional activation	Up-regulated	[218]
			Deleted Split hand/Split foot 1 (*DSS1*)	Hypomethylation of gene giving rise to high expression level of DSS1	Up-regulated	[219]
			Telomerase reverse transcriptase (*TERT)*	Hypermethylation of CpG islands leading to e inactivation of tumor suppressor gene	Down-regulated	[220]
			Ras association domain family 1 isoform A (*RASSF1A*)	Hypermethylation of promoter sites of this gene, which lead to cell-cycle development block from the G1 to the S phase	Down-regulated	[221]
			*P16INK4A(CDKN2A)*	Hypermethylation of P16INK4A is frequently associated with gene inactivation and the inhibition of CDK4/6	Down-regulated	[220]
			*BRCA1-associated protein-1 (BAP1)*	Hypermethylation leads to BAP1 loss leading to large-scale methylomic repatterning	Down-regulated	[220]
			The feline sarcoma *(FES)*	Hypomethylation of gene leading to melanocytic hyperproliferation	Down-regulated	[222]
			*P14ARF (CDKN2A)*	Hypermethylation of CpG islands leading to e inactivation of tumor suppressor gene	Down-regulated	[223]
			*PTEN*	Hypermethylation of gene suppressing the PI3K/AKT pathway	Down-regulated	[224]
			Retinoic acid receptor (*RAR*)-*β2*	Hypermethylation of specific tumor suppressor gene promoter	Down-regulated	[225]
			Microphthalmia-associated transcription factor (MITF)	Hypermethylation of gene leading to intrinsically low MITF expression	Down-regulated	[226]
	*Eraser*	DNMT or ten-eleven translocation (TET) methylcytosine dioxygenases	*TBC1D16*	Hypomethylation in metastatic melanoma tumor tissues	Up-regulated	[227]
	*Reader*	Methyl CpG binding proteins (MBPs)	*MAGE-A*	Hypermethylation of promoter has key role in restricting expression of the tumor-associated MAGE antigens	Down-regulated	[228]
*Histone Modifications*	*Writer*	Histone lysine methyltransferase (HKMTase)	Enhancer of zeste homolog 2 (EZH2)	Trimethylation of lysine 27 of histone H3 (H3K27me3) leading to transcriptional silencing of tumor suppressor genes	Up-regulated	[141]
			SET domain bifurcated 1 (SETDB1)	Trimethylation of lysine 9 of histone H3 (H3K9me3me3) leading to activation of thombospondin-1 (THBS1), and metastasis formation in melanoma	Up-regulated	[229]
			KMT2D	Monomethylation of lysine 4 of Histone H3(H3K4) deregulating particular promoter and genes in NRAS-mutant melanoma	Up-regulated	[230]
			EHMT	Mono/dimethylation of lysine 9 of histone 3(H3K9)	Up-regulated	[231]
		Histone acetyltransferases (HATs)	p300/CBP	Acetylation of lysine 27 of histone 3 (H3K27Ac)	variable	[232]
		Calmodulin-dependent protein kinase II (CaMKII)	cellular FADD-like, IL-β1-converting enzyme-inhibitory protein (c-FLIP)	Phosphorylation of c-FLIP upregulates its expression, thus making melanoma cells resistant to TRAIL-induced apoptosis	Up-regulated	[233]
		Protein arginine methyltransferases 1 (PRMT1)	Activated leukocyte cell adhesion molecule (ALCAM)	Arginine methylation of histones by PRMT1 regulating tumor growth and metastasis through targeting ALCAM	Up-regulated	[234]
	*Reader*	Bromodomain and extra-terminal domain proteins (BETs)	BRD2, BRD4	Acetylated lysine residues of histones are bound by BETs	Up-regulated	[235]
	*Eraser*	Histone Deacetylases (HDACs)	HDAC6	Deacetylation of related substares cause JAK/STAT3 and PD-L1 expression	Up-regulated	[236]
			HDAC1	Deacetylation of histones or non-histones substrates gives rise to increasing tumor cell growth	Up-regulated	[237]
			HDAC3	Deacetylation of histones or non-histones substrates cause increasing tumor cell growth	Up-regulated	[125]
			HDAC8	Deacetylation mediates adaptation of melanoma cells to multiple stress like BRAF inhibitor tolerance	Up-regulated	[151]
		Histone Demethylases (HMDs)	JARID1B (KDM5B)	Demethylates histone 3 at the position 4 lysine residue (H3K4)	Up-regulated (only in nevi)	[238]
			JMJD3 (KDM6B)	Demethylates histone 3 at the position 27 lysine residue (H3K27) changes microenvironment of melanoma tumorsand enhances tumor progression	Up-regulated	[238]
			LSD1 (KDM1A)	Demethylates histone 3 on lysine residues at positions 4 and 9 (H3K4 and H3K9)	Up-regulated	[239]
Non-coding RNA	–	snoRNA	miR-221	Sustaining proliferative signaling	Up-regulated	[84, 240]
			miR-193b	Sustaining proliferative signaling	Up-regulated	[84, 240]
			miR-449a	Cell cycle exit and epidermis differentiation	Up-regulated	[84, 240]
			miR-205	Enabling replicative immortality	Down-regulated	[84, 240]
			miR-18b	Resisting cell death	Down-regulated	[84, 240]
			miR-214	Activating invasion and metastasis	Up-regulated	[84, 240]
		lncRN	HOX transcript antisense RNA (HOTAIR)	Promoting proliferation of malignant melanoma cells via NF-κB pathway	Up-regulated	[241]
			SPRY4-IT1	Interacting with the PRC2 favoring the tri-methylation of H3K27 at specific target genes silencing of metastatic suppressor genes	Up-regulated	[209]
			Long:BRAF-activated ncRNA (BANCR)	Regulating both apoptosis and differentiation in melanoma	Up-regulated	[242]
			SAMMSON (survival associated mitochondrial melanoma-specific oncogenic lncRNA)	Maintainng oxidative phosphorylation and mitochondrial homeostasis	Up-regulated	[243]
			The metastasis‐associated lung adenocarcinoma transcript 1 (MALAT1)	Playing an oncogenic role in tumorigenesis via enhancing cancer‐cell proliferation, migration and invasion	Up-regulated	[244]
			Llme23	Playing an oncogenic role in human melanoma via direct binding to PSF	Up-regulated	[245]
Chromatin remodeling		The SWI/SNF (switch/sucrose non-fermenting) complex	ARID2, ARID1A ARID1B, SMARCA4, SMARCA2	Mutations in SWI/SNF components affecting its activity	Up-regulated	[246]
			BRG1	Microphthalmia associated transcription factor (MITF) and SOX10 actively recruit BRG1 to chromatin for establishing the epigenetic landscape of proliferative melanoma	Up-regulated	[246]
			ATRX	Interaction of ATRX with macroH2A to negatively induce its association with chromatin	Down-regulated	[247]
		NuRF chromatin remodeling complex	BPTF	Elevating BPTF expression has link to poor prognosis and acquisition of resistance to BRAF inhibitors	Up-regulated	[248]
		Polycomb repressive complex 2 (PRC2)	EZH2	Association with the beginning of a transcriptionally repressed state by the tri-methylation of H3 at lysine 27	Up-regulated	[249]
		Chromatin assembly factor-1 (CAF-1)	p60 subunit	Acting in strict association with both the S-phase and DNA repair processes	Up-regulated	[250]
Histone variants		MacroH2A	–	MacroH2A has ability of suppressing melanoma development through transcriptional repression of CDK8	Up-regulated	[251]
		H3.3	–	E2F target genes repression	Up-regulated	[252]
		H2A.Z.2	–	Promoting cell cycle progression	Up-regulated	[253]

### Chromatin methylation during melanoma

The methylation commonly occurs at the fifth carbon atom Cs in cytosine phosphate-guanine (CpG) dinucleotides [5]. DNA methylation is an essential epigenetic modification in many cancers [81]. The CpG dinucleotides are distributed in the human genome [82] that can be either as a dinucleotide or clusters as CpG islands. The CpG islands have some unique properties due to their localization in the promoter region of genes [83] and methylation in neoplastic conditions [84]. It is worth noticing that the hypermethylation of CpG islands has been mostly spotted in the promoter zones of the specific genes inducing the tumor suppressor genes silencing, chromatin remodeling and influencing the transcription, DNA repair, cell signaling, apoptosis, and cell cycle regulation of melanoma thus contributing to melanoma tumorigenesis [85]. In this line, DNA methyltransferases are the enzyme that adds methyl groups to the carbon atom of cytosine, culminating in the methylation of DNA [86]. In mammalian systems, DNA methylation is performed by DNMT1 and DNMT3s (DNMT3A and 3B). DNMT1 is predominantly involved in the maintenance of DNA methylation during cell division, while DNMT3s are involved in establishing de novo cytosine methylation and maintenance in both embryonic and somatic cells [87]. Thus, melanoma pathogenesis is developed owing to epigenetic alterations. Infinium methylation technology identified some CpG sites, associated with more than 14,495 cancer-related genes with significant methylation differences (44 hypomethylated and 106 hyper-methylated CpG islands) [88]. Some of the biomarker genes being modified through methylation are mentioned in Table 2.

The CDKN2A encoding for p16 tumor suppressor is either mutated or omitted in most melanoma cell lines. This gene could be transcribed in alternative reading frames, resulting in two separate gene products, p16 and ARF that both of which can negatively regulate cell cycle progression [52, 89–91]. The p16 exerts its effects by competitive inhibition of cyclin-dependent kinase 4 (CDK4) [92, 93]. So, p16 mutations increase the possibility of repair failure of DNA before cell division [94]. ARF is the second protein product of the CDKN2A locus, which regulates cell growth by affecting the p53 pathway [95]. The p16 mutation disables two separate pathways of cell growth control, which could indicate ARF role in cell growth [96, 97]. CDKN2A locus also encodes for the p14 protein, which binds to MDM2 and inhibits p53 ubiquitination and proteasomal degradation [97, 98]. Hyper-methylation of p14 has been shown in approximately 57% of human melanocytic nevi samples, while CDKN2A methylation has not been reported [99, 100]. On the other hand, hypermethylation of CpG islands could lead to modification of some genes such as the telomerase reverse transcriptase (TERT) gene, BRCA1-associated protein-1 (BAP1), microphthalmia-associated transcription factor (MITF), and Ras association domain family 1 isoform A (RASSF1A) [101–104]. Hypomethylation of specific CpG sites being close to the PRAME promoter and the deleted split hand/split foot 1 (DSS1) gene are other genes being regulated through DNA methyltransferases (DNMTs) [105, 106]. Overall, the identification of aberrant DNA methylation modifications in melanoma is considered a critical step toward comprehension and utilization of the methylation landscape in melanoma therapy [107].

### Histone modifications during melanoma development

Histone modifications are critical epigenetic drivers causing post-transcriptional modifications (PTMs) or altering the chromatin state proper for the cancer progression [108, 109]. Histones are characterized by positively charged and lysine-rich N tail regions [110]. Epigenetic modifications that happened mainly in tail domains can alter transcription and replication, or cause malignant transformations [5]. Multiple histone modifiers have been introduced and some of the histone modifiers are mentioned in Table 2. Ribosylation, phosphorylation, or histone ubiquitination has been involved in the regulation of various pathways in the development of melanoma.

The chromatin compaction and initiation of transcription are influenced by the phosphorylation of histones in mitosis and meiosis [111]. Additionally, histone phosphorylation of H1, H2B, and H3 can have a remarkable impact on DNA repair and gene regulation [112]. Both cancer development and dysregulation of oncogenic kinases are shown to result from an aberrant function of histone phosphorylation. However, modification of histone acetyl groups (via histone deacetylases (HDACs), histone acetyltransferases (HATs), methyl groups (via histone lysine methyltransferase (HKMTase), and histone demethylases (HMDs)) are of great importance and frequency [113].

#### Modification of histones acetyl groups

**Fig. 1:**
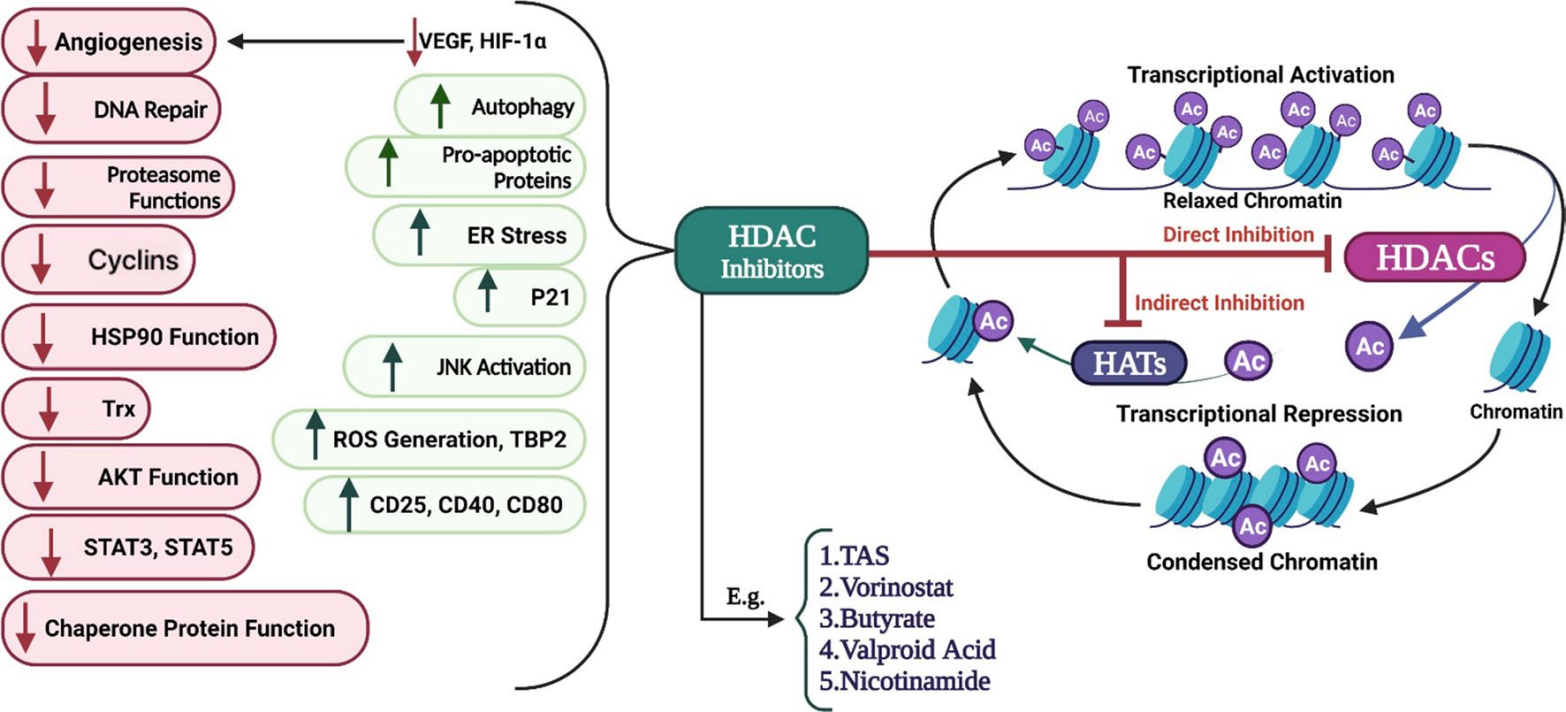
HDACs remove acetyl groups from chromatins, which can be inhibited by HDACis. They act based on two strategies, including the direct inhibition of HDACs or indirect inhibition of HATs. Irrespective of strategy, HDACs are used to treat melanoma because they can induce various mechanisms leading to the prevention of tumor cells to develop, namely decreasing cyclins, AKT function, angiogenesis, and so forth. Additionally, HDACs increase autophagy, pro-apoptotic proteins, P21, ER stress, JNK activation, CD25, CD40, and CD80

The regulation of chromatin structure and remodeling can be triggered by both acetylation and deacetylation, which have been considered as post-translational modifications (PTMs) identified in different developmental steps during cancers [109]. Histone acetyltransferases neutralize the positive charge of histones and diminish the tight-binding between the negatively charged DNA and the histone [114]. This acetylation changes a closed heterochromatin structure into open chromatin, thus promoting greater chromatin accessibility and gene transcription [115]. Conversely, histone deacetylases change open chromatin to closed one and lead to the prevention of gene expression [115]. Histone deacetylase activity within the promoter region of cancer-related genes can lead to cancer [5]. In the case of melanoma, histone deacetylases (HDACs) regulate the MAPK pathway. Therefore, it could affect the cancer progress and modulate the response to anticancer drugs (Fig. 1).

The HDACs are well-studied histone modifier enzymes obliterating acetyl groups on the histone tails. They have critical roles in signaling pathways driving melanoma pathogenesis [116]. Moreover, HDACs are capable of modifying other proteins that lack any association with the chromatin environment [117, 118]. Many reports have highlighted the role of HDAC inhibitors (HDACi) in the prevention of tumor cells from proliferating excessively through various mechanisms [119]. Although HDACi approved by FDA have been shown to have CR for treating cutaneous and peripheral T-cell lymphoma (CTCL and PTCL), the efficacy of these molecules remains to be fully elucidated [120].

In addition, the expression of programmed death-1 / programmed death-ligand 1 (PD-1/PD-L1) and the genes with crucial functions in immune evasion are regulated by HDACs [121, 122]. It has been demonstrated that HDACs can reversibly deacetylate the lysine residues in local histones; as a result, they could decline the expression of tumor suppressor genes in the case of melanoma [123]. Booth et al. examined the therapeutic behavior of HDACi in melanoma cells [124]. They proposed a melanoma treatment with HDAC inhibitors. These inhibitors could rapidly diminish the expression level of various HDAC proteins, PD-L1, PD-L2, and ornithine decarboxylase (ODC). They also could increase the expression of Major Histocompatibility Complex Class I A (MHCA) through modulation of HDAC1, HDAC3, HDAC8, and HDAC10, and decrease the expression of PD-L1, PD-L2, and ODC on melanoma tumor cells. These properties indicate that pan-HDAC inhibition usage could be more impressive than a specific HDACi. Previously, it was reported that the lethality of pazopanib could be significantly enhanced via knockdown of [HDAC6 + HDAC2] or [HDAC6 + HDAC10], while the knockdown of [HDAC6 + HDAC1] or [HDAC6 + HDAC3] is less effective in melanoma cells [125]. Emmons et al. revealed that HDAC8 causes transcriptional plasticity in melanoma cells through direct deacetylation of c-Jun [126]. Other anticancer histone deacetylase inhibitors like valproic acid, trichostatin A, panobinostat, tenovin-6, and other HDACIs have been reported in melanoma by Moschos and Yeon [121, 127]. Altogether, four HDAC inhibitors approved by FDA are Vorinostat (hydroxamic acid family), Romidepsin (cyclic peptide family), Belinostat (hydroxamic acid family), and Panobinostat (hydroxamic acid family), which are prescribed in lymphoma patients [128]. Despite various researches, no prosperous clinical trials involving HDACIs (even alone or in combination with immune checkpoint inhibitors) have been reported in melanoma cases yet. The expression levels of immune checkpoint molecules can also be regulated by HDACs, whereby this regulation is as an attractive method to dominate the immune checkpoint blockade resistance in the treatment of melanoma [129, 130]. Unfortunately, the currently in use HDAC-selective inhibitors have off-target effects highlighting the need to improve the potency and selectivity of the HDAC inhibitors. This purpose could be achieved by HDAC-specific inhibitor design according to their unique structures [131, 132]. The identification of agents being capable of binding with individual HDACs could be helpful in the introduction of new anti-melanoma therapies. On the other hand, identifying the complicated HDAC biology and unique cellular toxicity profile of HDACIs will allow the recognition of the most suited patient population for HDACI based treatments [133]. In addition to the importance of HDACs discussed above, gaining insight into the significance of histone acetylation for melanoma development has been disclosed using a zebrafish model. Kaufman et al. developed a triple transgenic zebrafish model (p53/BRAF/crestin: EGFP) to investigate molecular events beyond genetic changes causing melanoma progression [134]. Melanomas can reestablish the crestin: EGFP expression, which indicates the ability of these cells to revert into a neural crest progenitor state [135]. SOX10 expression is regulated by the acetylation of lysine 27 on histone 3 (H3K27Ac) and somatic inactivation of two subunits of the NURF complex (Brg1 and Bptf). Thus, SOX10 dictates fundamental gene expression programs in melanoma cases. Notably, co-regulation of both transcription factors and chromatin remodeling with MITF and SOX10 also can result in the dictation of gene expression programs [135].

The other crucial histone acetylation process is the identification of chromatin modification by “reader” proteins, which leads to the initiation of downstream regulatory processes. The bromodomain and extra-terminal domain (BET) proteins BRD2, BRD3, BRD4, and BRDT bind to acetylated lysine residues of histones and other master transcription factors to regulate gene expression. Both BRD2 and BRD4 are imperative for the maintenance of tumor cells which are overexpressed in the cases of melanoma compared to other BETs [116]. Gallagher et al. have introduced a bromodomain inhibitor with the following features: 1) selective inhibition of cell cycle. 2) inhibition of pro and anti-inflammatory genes such as NF-κB, VEGF, and CCL-20. 3) downregulation of IL-6 and IL-8 production through BRD2 displacement. 4) excitation of caspase-dependent apoptosis [136]. BrDi was shown to bind effectively with BET family members. Interestingly, it has been shown to have a cytostatic impact and G1 arrest properties. It is noteworthy that key cell cycle genes (SKP2*, *ERK1, and c-MYC) and accumulating cyclin-dependent kinase inhibitors (p21 and p27) are downregulated by BET displacement [137]. These findings suggest using BET family inhibitors instead of BrDi would deteriorate in vitro and in vivo melanoma cell growth. Furthermore, based on transcriptomic analysis of melanocytes exposed to the BET inhibitor JQ1, a transmembrane protein named AMIGO2 was identified as a BET target gene crucial for melanoma cell survival [138].

#### Modification of histones methyl groups

**Fig. 2:**
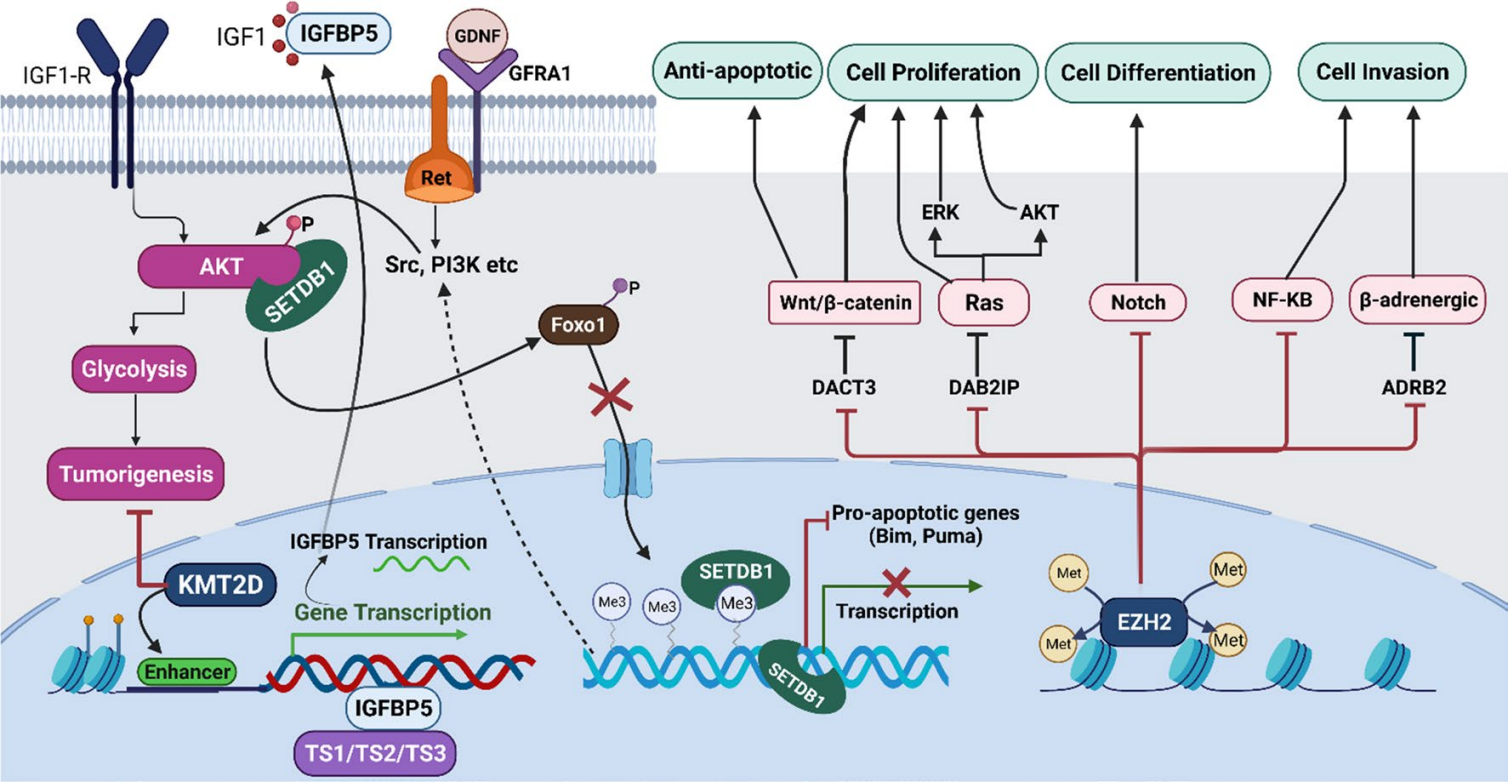
Histone lysine methyltransferases, namely KMT2D, EZH2, and SETDB1 can affect the advancement of melanoma cases. KMT2D can act as a regulator of IGFBP5 transcription to repress IGF1, thus inhibiting tumorigenesis. The SETDB1 can be triggered by Ret as a result of Src and PI3K pathway activation thenceforth, which affects AKT binding with SETDB1. This event leads to inhibiting pro-apoptotic genes such as Bim and Puma, and transcription blockade. EZH2 is involved in sundry signaling pathways such as Wnt/β-catenin, Ras, Notch, NF-KB, and β-adrenergic that deregulation of which can lead to tumorigenesis

Histone methylation that is triggered by histone methyltransferases (HMTs) has critical role in the adjustment of gene transcription and is essential for chromatin remodeling [139]. Histones are methylated on arginine or lysine residues [140]. The position and degree of methylation (number of methyl groups added) are essential in this regulation [141]. For instance, trimethylation at lysine 9 (K9) of histone H3 leads to closed chromatin structure and silencing of the related genes. At the same time, demethylation and mono-methylation in the same position form the opened chromatin structure and activate the corresponding genes [5, 142]. Depending on the position of the modified residue, the methylation of histone can both suppress (H3K27, H3K9) and elevate (H3K4) the gene expression [143]. Demethylase plays a critical role in the disease progression and drug resistance in the case of melanoma [144]. Histone lysine methyltransferases, namely KMT2D, SETDB1, and EZH2, are the large classes of enzymes that catalyze site-specific methylation of lysine residues on histones and other proteins, playing significant functions to control transcription, chromatin architecture, cellular differentiation, and melanoma progression (Fig. 2).

It is worth mentioning that gene expression silencing can be controlled by the H3K9me3-specific histone methyltransferase SET domain bifurcated 1 (SETDB1), which is catalyzed by the methylation of lysine 9 on the histone 3 [145]. Most importantly, SETDB1 is amplified among the cases of human melanoma in comparison with nevus or normal skin. Moreover, it can exacerbate tumor cells in animal models, known as an encouraging therapeutic target in melanoma [146]. Orji et al. unraveled that SETDB1 may act on regulating H3K9me3 distribution and add epigenetic marks such as activation of thrombospondin -1 (THBS1) [142]. The EZH2, H3K27me3-specific histone methyltransferase enhancer of zeste 2 polycomb repressive complex 2 subunits, is the other deregulated histone-modifying enzyme during the melanoma initiation and progression. Increasing the level of both EZH2 and H3K27me3 have been reported in aggressive melanoma cell lines, whereby tumor suppressors RUNX3 (RUNX family transcription factor 3) and E-cadherin expression are suppressed via enabling senescence evasion [147]. The capability of EZH2 to recruit histone deacetylases was found by some studies, hence showing functional synergy with H3K27me3 to silence genes [148]. Based on reports, the non-canonical NF-kB pathway could be regulated by EZH2 expression through NF-kB2 direct binding with the EZH2 promoter. Therefore, pharmacological inhibition of EZH2 is an engrossing target in various cancers including melanoma [149–151]. The other new epigenetic mechanisms that have been detected are exerted by KDM6B. This protein triggers epigenetic mechanisms by upregulating various targets of both NF-κB and BMP (Bone Morphogenic Protein) signaling to exacerbate the emigration of melanoma cells provoking tumor cell metastasis [152]. It should be noted that inhibition of the MAPK pathway reduces the H3K4me3 and H3K9ac in the mutant TERT promoter region. These changes result in a noticeable decline in TERT transcription and RNA polymerase II (Pol II) recruitment. Notably, TERT transcription can be activated by binding between ERK2 with TERT promoters and inhibiting the HDAC1 repressor complex recruitment [153]. As shown, reduction of histone acetylation marker such as H3K27Ac, H2BK5Ac, and H4K5Ac can characterize chromatin state transitions. Moreover, di-/tri methylation of H3K4 is strongly linked with melanoma being relevant to signaling pathways such as PI3K, IFNγ, LKB1, TRAIL, and PDGF. Therefore, epigenetic alterations and melanoma progression are highly correlated [116].

### The Role of micro RNAs /noncoding RNAs in pathogenesis of melanoma

**Fig. 3:**
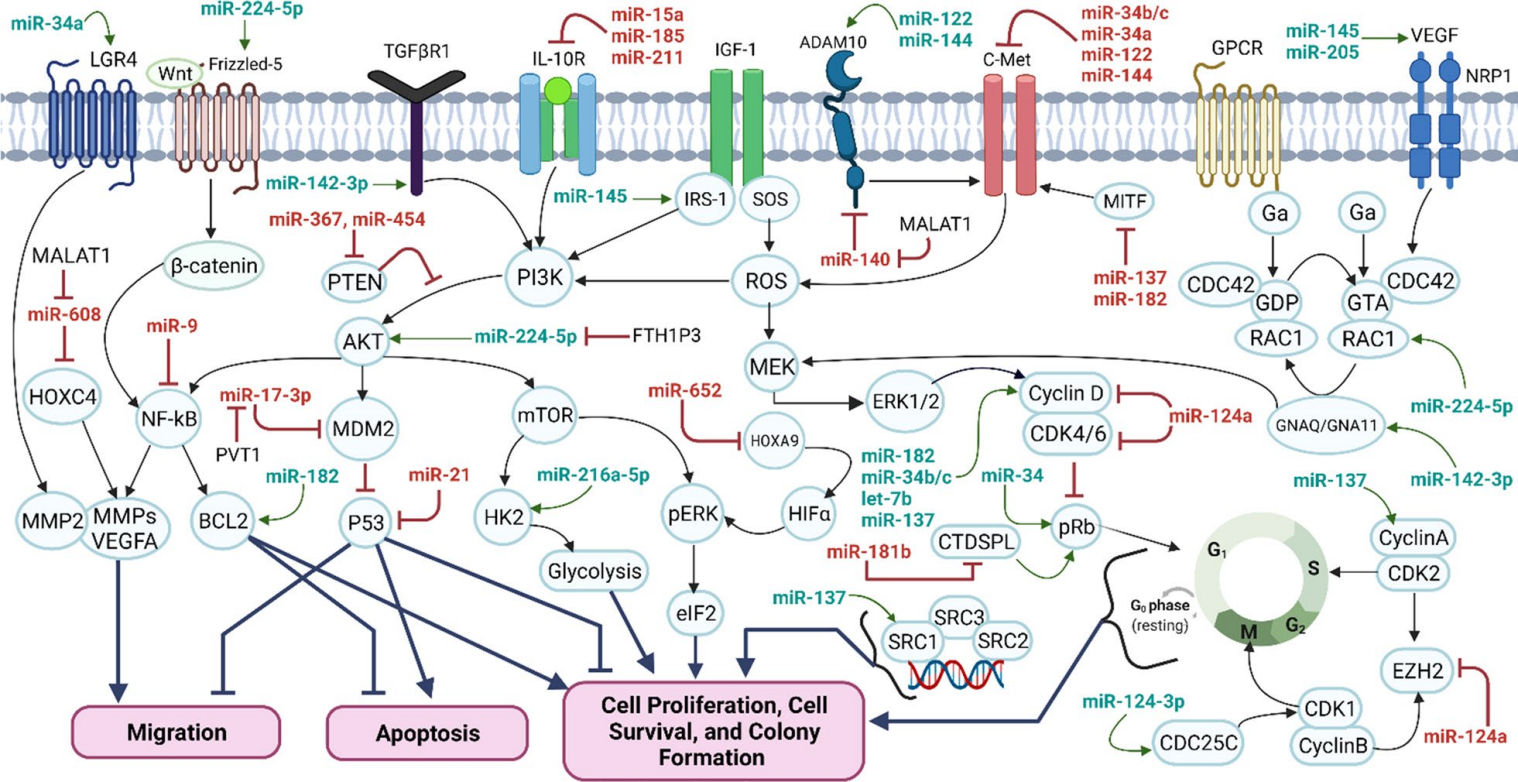
Some of the miRNAs affect migration, apoptosis, proliferation, and survival of melanoma cells via various mechanisms

MicroRNAs (miRNAs), single-stranded short noncoding RNAs, play a vital role in expressing nearly 60 percent of human protein-coding genes that their dysregulations lead to several disorders such as cancer [55, 154]. In Melanoma cancer, miRNAs are involved in numerous cellular events, including melanoma genesis, cell cycle regulation, tumor growth and proliferation, cell migration and invasion, drug resistance, and apoptotic induction. Accordingly, the biological processes of melanoma cancer cells are potentially affected by downregulated miRNAs, including miR-211, miR-196a, miR-21, miR-124, miR-29c, and miR-210 [155, 156]. Interestingly, miR-211 has been identified as differentially expressed in the melanoma cell lines among various types of miRNAs affecting numerous targets like TGFBR2 (transforming growth factor beta receptor 2), RUNX2, IGF2R (insulin like growth factor 2 receptor), and NFAT5 (nuclear factor of activated T cells 5). Moreover, the ectopic expression of miR-211 involves the inhibition of migration and invasion in melanoma cells. This property suggests the tumor suppressor activities of miR-211 [157, 158]. Notably, the expression of miR-196a, miR-200c, and miR-205 could lead to extensive down*-*regulation of malignant melanoma cell lines and act as the tumor suppressors [159]. In contrast, numerous miRNAs such as miR-210, miR30b, and miR-30 are overexpressed in melanoma and associated with up-regulation of cancer cells leading to melanoma metastasis through promoting invasion and immunosuppression induction [160]. MiR-149 is another overexpressed miRNA in melanoma targeting GSK3a leading to apoptosis resistance in melanoma cells. So, non-coding RNAs (mainly miRNA) deletion can/state control both normal and melanoma cells generally affecting cell cycle regulation [161]. For instance, let-7b is a type of miRNA that inhibits the cell cycle progression by decreasing the CCND1, D3, and CDK4 expressions and it functions as a cancer cell growth [162]*.* Moreover*,* miRNA-193b downregulates CCND1 and CCND2 genes, which results in the promotion of melanoma cell proliferation and invasion [163]. Downregulation of miR-206, miR-143, or miR-106b inhibits *CCND1* via affecting G1 cell cycle causing decrease in melanoma cells invasion or migration. Several other miRNAs have been documented as the significant cell cycle regulators in a cyclin-independent manner, including the miR21, miR203, miR205, miR18b, miR149, and miR26a [164]. Apoptosis induction by some microRNAs, including miR-155, miR-205, miR-21, miR-26a, miR-15b, and miR-149 have been highlighted in various reports[165]. Alteration in methylation of CpG islands regulate the expression of miR-375, miR-34b, miR-182, miR-148a, miR-203, miR-29, and miR-26 demonstrating the epigenetic regulation of miRNAs in melanoma [166]. As aforementioned, some miRNAs function as anti-melanoma in combination with HDACIs and immune checkpoint inhibitors. For example, downregulated miR-589 promotes melanoma malignancy through accelerating PD-L1 expression level [167]. Further, combinatory inhibition of a miR-146a and PD-L1 promotes the survival in a melanoma mouse model [168]. Although the down/upregulation of miRNAs in melanoma cells and the consequences of such dysregulations have been vastly reported. Although, the related mechanisms of these dysregulations are not entirely understood. Some of the miRNAs affecting apoptosis, migration, and proliferation of melanoma cells through different mechanisms are presented in Fig. 3. Overall, miRNAs can be beneficial in scientific research, the diagnosis of melanoma, and also it can be used to predict the patient’s reactions to treatments. Therefore, the development of miRNAs is considered as critical epigenetic factor in melanoma which could greatly increase the clinical management of melanoma.

## Epigenetic impact on drug resistance in melanoma

Differences among patients are the key reason which leads to different effectiveness and toxicity of treatments and specific roles that are played by drugs. It is of note that some features of medicines such as their uptake, process, and metabolism in specific tumor cells are distinct among different patients who have sundry genetic/epigenetic underlying [169]. It should also be noted that tumor cells are capable of tolerating drug impacts via enhancing new molecular mechanisms and activating alternative compensatory pathways to bypass the treatment effects. Hence, gaining knowledge about the mechanisms and pathways triggered by anticancer drugs seems imperative [170].

Epigenetic alterations have also been shown to rewire the chromatin landscape of melanoma cells to tolerate the current therapies. This event is because the chromatin-mediated alterations are shown to be reversible [171]. In another study, Sharma et al. identified tumor cells that survived in the presence of a 100-fold drug concentration more than the IC50 used in other tumor cell lines [172].

Several studies have found that H3K4me3/2 histone modification can be declined by a histone demethylase named KDM5A (JARID1A). KDM5A is vital for the reversible drug-tolerant state due to the RNAi-mediated knockdown. The expression of this enzyme was found in BRAFi treatment which is relevant to the drug-tolerant state phenotype [172]. Accordingly, induced drug-tolerant cells (IDTCs) can occur under external stress conditions, namely hypoxia and nutrient starvation. This event leads to increased expression of both the H3K4 demethylases (KDM1B, KDM5A, and KDM5B) and the H3K27 demethylases (KDM6A and KDM6B) [173]. In addition, a noticeable decline in the number of melan-A and tyrosinase (as differentiation markers and MITF target genes) has been reported in IDTCs. Hence, the cell transition into an undifferentiated state pertains to elevated aggressiveness [174]. Therefore, it could be deduced that histone demethylases and cancer cell dedifferentiation have essential roles in the epigenetic regulation of distinct drug resistance mechanisms following BRAF inhibitor treatments. These effects are exerted via chromatin remodeling mechanisms, including either loss or gain of histone post-translational modifications [175]. Shalem et al. found that the STAGA HAT complex members (TADA2B and TADA1) can induce histone acetylation when melanoma drug resistance has occurred [176]. Recently one study in melanoma indicated that inhibition of SIRT1 declines melanoma cell growth and increases their sensitivity to PLX4032 [177]. Moreover, SIRT2 inhibition showed the resistance of melanoma cells to MAPKi via ERK reactivation [178]. CRISPR-Cas9 screen method in drug resistance melanoma has also revealed that chromatin-associated with histone deacetylase SIRT6 can be considered a regulator of resistance to BRAFi (dabrafenib) and BRAFi + MEKi (dabrafenib + trametinib). HATs such as KAT1 (HAT1) and KAT2B (PCAF) have been identified to confirm the epigenetic impact in melanoma [179, 180], So that increasing the activation of the AKT signaling pathway gives rise to MAPKi resistance [181, 182]. This resistance can be triggered by IGFBP2, as it is a part of a gene signature to respond to MAPKi “drug-tolerant persisters” [183]. Notably, both MAPK and IGF-1R pathways in tandem with each other can block/delay resistance processes to targeted MAPKi therapies, specifically for cases with great levels of IGFBP2.

It also was reported that a high level of lipogenesis is a noticeable metabolic characteristic of cancer cells for membrane biogenesis and energy metabolism. Accordingly, ACLY expression, the critical rate-limiting enzyme in lipogenesis, is significantly increased in melanoma as an oncogenic factor. This enzyme regulates the MITF–PGC1a transcriptional axis to enhance melanoma growth. In this line, ACLY increases histone acetylation at the MITF locus, and facilitates transcriptional activation of the MITF–PGC1a axis. Therefore, the combination of MAPK and ACLY can be efficient in melanoma treatment [184].

Another concern is the acquisition of resistance to alkylating agents that are effective in 10–20 percent of cases in monotherapy [185]. These agents can dump the cells to death via binding to DNA. Specific DNA repair machinery types such as mismatch repair (MMR) will therefore recognize modified nucleotides. That is why activated DNA repair enzymes such as O6 -methyl-guanine-DNA methyltransferase (MGMT) decrease the drug effects [186]. Esteller et al. have found that 40 percent of gliomas treated with alkylating agents lead to MGMT inactivation by hypermethylation in its promoter [187]. This finding also was shown in other tumor types, including glioblastoma, melanoma, and colorectal cancer [188]. In melanoma patients, reactivation of MGMT was demonstrated via hyper-methylation or SNPs in its corresponding gene. Hence, fotemustine resistance and more tolerance to temozolomide treatment could occur [189]. Additionally, the expression level of proteins involved in DNA damage recognition and its repair can be associated with resistance to alkylating agents [190].

## Epigenetic impact on targeted therapy efficiency in melanoma

Since the epigenetic marks are reversible and targeted immunotherapies are adaptable and widespread, numerous anticancer strategies to rebalance the epigenome return to the normal state are under development. As previously mentioned, drug holiday is one of the most common concepts of non-genetically regulated drug resistance. For example, it was reported that patients re-treated with BRAF or BRAF/MEK inhibitors had shown great responses [171]. Moreover, intermittent dosing schedules can lead to the delayed occurrence of vemurafenib resistance in melanoma xenograft mouse models, compared to those who commit to continuous treatment [191]. Nevertheless, studies carried out regarding vemurafenib sensitivity showed that chromatin assembly factor 1 (CAF-1) plays an essential role in retaining vemurafenib sensitivity. To verify the CAF-1function, it has been demonstrated that obliteration of CAF-1 gives rise to a diminution in genome-wide H3K9me3 and BRAFi resistant cells. The CAF-1 can therefore facilitate the integration of H3-H4 tetramers at the DNA replication fork during the S phase of the cell cycle, and lead to H3K9 methylation [192]. It is worth mentioning that a high level of KDM5B could lead to drug resistance. Thus shRNA-mediated knockdown of KDM5B gene can increase the sensitivity of different drugs [173]. Owing to the dynamic features of KDM5A and KDM5B, long-time exposure to external stressors could lead to an innate cellular response and hence a multidrug-resistant phenotype [174]. Continuous exposure of melanoma cells to IDTCs makes them unresponsive to the 20-fold dose of taking BRAFis, MEKis, trametinib, and cisplatin. Additionally, IDTCs (hypoxia and nutrient starvation) retrieve drug sensitivity after seven days of ceasing drug activation.

It has been shown that the expression of melanoma stem cell markers including CD44, NGFR, SOX10, SOX2, and SOX4 was increased by IDTCs. The expressions of ABCB5, ABCA5, ABCB8, and ABCB4, were escalated likewise. These events give rise to an undifferentiated state [174], and can affect histone marks; for instance, H3K4me3 and H3K27me3 are diminished, while H3K9me3 is increased [174]. These observations suggest that drug-independent generic stress responses can be regulated epigenetically in environmental conditions [174]. These findings implicate strategies, which target the slow-cycling drug-tolerant phenotype, which will be beneficial. Sharma et al. demonstrated that HDACIs have a noticeable impact on the subpopulation with high KDM5A expression, which is appeared after exposure to large drug concentrations [172]. This observation results from the link between KDM5A, and histone deacetylates during the removal of methylation patterns for lysine 4 and 9 on histone 3 [193]. Moreover, HDACIs induce apoptosis, while the combination of HDACs could lead to improved drug resistance in the subpopulation [172]. Roesch et al. have indicated that enrichment of the cells with high KDM5B expression during drug treatment of melanoma cells is dependent on high levels of expression for oxidative phosphorylation enzymes of the electron transport chain, such as ubiquinol cytochrome c reductase, NADH dehydrogenase, cytochrome c oxidase, and ATP synthase [173]. Therefore, inhibition of the mitochondrial respiratory chain via rotenone, oligomycin, or phenformin can decrease KDM5B expression, and subsequently, drug resistance. Noteworthy, a remarkable decline in drug resistance was shown due to combination therapy with phenformin (NADH dehydrogenase inhibitor), vemurafenib, and BRAF inhibitor [194]. According to obtained evidence, the expression of endogenous PGC1a depends on the growth of the mitochondrial function. Moreover, it is tolerant of oxidative toxicity in a subset of melanomas. Therefore, mitochondrial homeostasis is critical in the progress of melanoma. The MITF is shown to be highly expressed in melanoma cases. MITF manages the transcription of PGC1a and mitochondrial biogenesis. It is of note that BRAF is capable of inhibiting MITF–PGC1a axis of transcription and subsequently the mitochondrial function. Simultaneous expression of both MITF and PGC1a and inhibition of BRAF or MEK could promote oxidative phosphorylation. Therefore, inhibition of MITF–PGC1a axis and mitochondrial function is a potential therapeutic strategy to avert melanoma development and boost the efficacy of MAPK inhibition [184, 195].

The IDTC phenotype of melanoma cells is not susceptible to certain treatments, such as a combination of BRAF inhibitors with oligomycin, HDACIs, and AKT inhibitors [174]. For instance, KDM5B gene elimination sensitizes melanoma cells to BRAF blockage, despite the fact that survived cells display the IDTC phenotype. Taking different drugs such as MEK, AKT, and HDAC inhibitors in IDTCs condition to suppress their target pathways over three days of therapy was shown to be remarkably effective. However, adaption to these drugs of melanoma cells can be seen after 12 days of treatment [196]. Nonetheless, 90 days of exposure of melanoma cells to BRAF inhibitors displays no multidrug resistance, leading to the elimination of IDTC markers such as NGFR and KDM5B and permanent resistance [174]. According to what has been discussed so far, multiple epigenetic changes, mainly histone modifications, variation in miRNA expression levels, and hypo/hypermethylation of oncogenes or tumor suppressor genes, are well characterized to be related to melanoma tumorigenesis like many other cancers. Further studies are expected to elucidate the generation and regulation mechanisms of these epigenetic changes in the development of cancer cells.

## Combination of immunotherapy compounds with epigenetic drugs in melanoma

Cancer cells bypass the immune system via different epigenetic mechanisms. Downregulation of genes, which are involved in the presentation of tumor antigens, is an epitome of such mechanisms. In this regard, many pharmacological agents/therapies have been developed to inhibit these mechanisms and reprogram post-translational histone modifications [197]. It also was found that immune or inflammatory-related genetic factors have been escalated due to the combination of these therapeutic agents with immunotherapy during the inhibition of the epigenetic mechanisms.

### Epigenetic drugs based on histone and/or chromatin modifications

Based on reports, cancer patients treated with either HDACIs, DNMT, or PD1/PD-L1 immune checkpoint inhibitors experienced potent treatment responses. These studies suggest that these epigenetic inhibitors may escalate the efficacy of immunotherapy via [1] enhancing the antigenicity, [2] counteracting immunosuppressive mechanisms by the tumor microenvironment, and [3] reversing cytotoxic T cell exhaustion [198]. It is reported that patients suffering from melanoma with the expression of PD-L1 are divided into four groups based on the number of tumor-infiltrating lymphocytes (TILs). Group 1 patients respond to treatment, which is responsive against their tumor cells. Group 2 patients have a low number of TILs and negligible or no PD-L1 expression, thereby not responding to PD1 monotherapy. Group 3 patients have TILs, albeit low or no PD-L1 expression. Eventually, Group 4 patients who have few or no TILs, albeit having PD-L1 expression [138, 139]. It is of note that PD-L1 expression can be increased in epithelial cancer cell lines, which are under treatment with DNMT inhibitors (DNMTis). Moreover, Illumina 450 K arrays revealed that low or no PD-L1 expression is strongly linked with high DNA methylation. This property reveals the role of chromatin methylation to suppress PD-L1 expression. Transcription factors or epigenetic regulators including the EZH2 and SUV39H1 methyltransferases are capable of direct interaction with binding domains (ATRX-DNMT3-DNMT3L (ADD)) of DNMT3A. Therefore, DNA hypomethylation regulates the PD-L1 expression, and it plays a pivotal role in the modulation of responsiveness to PD1 inhibitors [199]. DNA methyltransferase 1 (DNMT1) locates on the daughter strand cytosine at the complementary CpG. This process enforces gene silencing simultaneously to the mammalian cell division [200, 201]. DNA methyltransferase inhibitors (DNMTis), namely 5-azacytidine, decitabine, and guadecitabine, are approved for DNA histone hypo-methylation in patients suffering from the myelodysplastic syndrome or leukemia to reactivate tumor suppressor genes. These Aza nucleosides irreversibly bind to DNMT1 and degrade them by substituting nitrogen with carbon at the C-5 position of the pyrimidine ring. This substitution results in the loss of DNA methylation, expression of genes pertaining to immunomodulatory pathways, and induction of tumor antigen presentation [202]. For instance, NSCLC cell lines under treatment with 5-azacytidine can activate the JAK/STAT signaling pathway and provoke the expression of genes with a role in antigen presentation. This event per se culminates in the expression of PD-L1, considered a vital ligand-mediator of immune tolerance. Moreover, DNMTi has been shown to be linked with the activating of expression for hyper-methylated endogenous retroviral double-stranded RNAs (EVs). This property of DNMTi could lead to the induction of type I interferon response and MHC I expression [203]. Researches in tumor-bearing mice models indicated that previous treatment with decitabine (as a DNMTi) affects either the tumor cells or antigen-specific CD8 + T cells. This treatment accompanied by anti-PD-L1 agents can prevent the acquisition of exhaustion-associated methylation programs. This event makes the T cells become more potent for expansion after immune checkpoint blockade [204, 205]. In a murine ovarian cancer model, anti-CTLA-4 treatment became more potent via its combination with decitabine. This combination increased the differentiation of naive T cells into effector T cells [205].

### Epigenetic drugs based on ncRNAs suppressing/activating

Aside from the aforementioned, non-coding RNA molecules (ncRNAs), miR-125a, miR-28, miR-125b, miR-100, miR-200c, miR-211, MELOE, SAMMSON, and HOTAIR have been found to play a crucial role in treatment resistance [206]. miRNAs promote the melanoma to secondary sites via several mechanisms, including (A) regulation of MITF-M expression, (B) alteration of the extracellular matrix (ECM), (C) enhancement of reciprocal epithelial-to-mesenchymal transition (EMT), mesenchymal-to-epithelial transition (MET), and (D) preparation of pre-metastatic niche formation [207]. However, some mRNAs such as miR-182, miR-137, miR-211, and miR-107 reduce the MITF-M expression in melanoma cells, leading to an invasive phenotype [208]. For instance, melanoma cell invasion and migration are provoked as a result of miR-182 upregulation which is triggered by the downregulation of both expressions of MITF and FOXO3 [158]. On the other hand, miR-211 can block the invasion and migration of melanoma cells [156], and repress POU3F2 (POU-domain class 3 transcription factor 2, also known as brain-specific homeobox 2 (BRN2)) which acts as a MITF suppressor. Zhao et al. have indicated that downregulation of miR-107 (a tumor suppressor) represses melanoma cell invasion through POU3F2 targeting [209].

miRNAs have conflicting functions, such as enhancing either tumor migration or suppression. For example, the miR-224/miR-452 cluster is directly activated by E2F1, which facilitates the cytoskeletal rearrangement of less aggressive cells and thereby increases the migration and invasion of melanoma cells. Notably, the miR-200 family (miR-200a, miR-200b, miR-200c, and miR-141) induces EMT-like processes via upregulation of Bmi-1 oncogene expression. Thus, the PI3K/AKT and MAPK pathways can be activated. Activation of these pathways negatively impresses the expression of ZEB1 (zinc finger E-box-binding homeobox 1) and E-cadherin, which provokes the expression of vimentin and N-cadherin [209]. Pertaining to IncRNA effects on melanoma cells, it is of note that SPRY4-IT1 was the first lncRNA, which was characterized to be originated from an intron of the SPRY4 gene. Recent studies have found that the expression of SPRY4-IT1 is escalated in cases of melanoma [210]. Siena et al. have identified a remarkable upregulation of ZEB1 antisense RNA 1 (ZEB1-AS1) in metastatic melanoma linked with hotspot mutation in both BRAF and RAS family genes [211]. Their analysis showed that ZEB1-AS1 could function by activation of expression for zinc finger E-box binding homeobox 1 (ZEB1). Activation of EB1 could influence the invasiveness and phenotype switching melanoma cases [211]. GAS5 is a lncRNAs, which diminishes the expression of MMP2. This protein is involved in ECM degradation and can reduce the migration and invasion of human MM cells [212, 213]. Nonetheless, deregulation of the expression for some miRNAs could give rise to drug resistance, particularly in BRAFi or MAPKi-based melanoma therapies. As a good example, miR-31a, miR-100, and miR-125b are shown to stimulate tumor cell proliferation, apoptosis escape, and decline in drug sensitivity among patients who took vemurafenib. Additionally, inhibition of miR-125a causes drug re-sensitization in a subset of BRAFi-resistant cell lines of melanoma. miR-204 and miR-211 could also lead to resistance against vemurafenib in melanoma cells [214–218].

## Epigenetic drugs in clinical trial to treat melanoma

Histone deacetylases are known to play a pivotal role in the transcriptional machinery for regulating gene expression, inducing histone hyperacetylation, and affecting gene expression. Therefore, they represent the target of therapeutic or prophylactic agents, HDACis, for diseases caused by abnormal gene expression.

HDACi have manifold biologic effects resulting from alterations in patterns of acetylation of histones and many nonhistone proteins, which include proteins involved in the regulation of gene expression, cell cycle progression, pathways of extrinsic and intrinsic apoptosis, redox pathways, mitotic division, angiogenesis DNA repair, and cell migration.

Valproic acid (VPA) and pivaloyloxymethyl butyrate (Pivanex, AN-9), two short-chain fatty acids, are supposed to be use in the treatment of melanoma. Phase I/II clinical trials tested VPA alone or in combination treatment for melanoma, and the conclusion was that VPA potentiates KTN-induced DNA strand breaks and cytotoxicity [255]. VPA also was examined in another phase I/II clinical trial which combined with standard chemoimmunotherapy in patients with advanced melanoma. On the contrary, the combination of VPA and chemoimmunotherapy did not produce results overtly superior to standard therapy [256]. Two serious adverse events stemmed from taking VPA—a grade 3 neurological toxicity and a grade 4 bleeding of a cerebral metastasis—were shown in this study [256]. Pivaloyloxymethyl butyrate (Pivanex, AN-9) is the other short-chain fatty acid that is in phase I/II clinical trials for malignant melanoma, and AN-9 exhibited antimetastatic and antiangiogenic activities via decreasing vascularization, bFGF expression, and HIF-1α [257]. Mild to moderate nausea, vomiting, hepatic transaminase elevation, hyperglycemia, fever, fatigue, anorexia, injection site reaction, diarrhea, and visual complaints were side effects observed in sundry studies in the treatment of patients afflicted with solid malignancies [258].

Benzamides are a class of drugs composed of HDACi containing a characteristic 20-aminoanilide moiety able to contact specific amino acids in the tube-like active site of the HDAC core, with or without coordination/chelation of zinc ions [259]. MS-275 (SNDX-275, Entinostat) is a class I selective inhibitor of benzamides in phase II in patients with melanoma in a clinical trial with NCT00185302. Reported dose-limiting toxicities associated with entinostat include neurotoxicity, fatigue, hypophosphatemia, anorexia, and vomiting [260].

## Conclusion and future directions

Chromatin remodeling, histone modifications, DNA methylation, and microRNAs are considered as epigenetic mechanisms, which could be exploited to predict treatment outcomes via regulating the expression of several functional genes. This review has abridged a notable topic regarding the genetic and epigenetic changes that have remarkable roles in the enhancement and progression of melanoma. Cancer-based researches are about changes from a histology-based standpoint in genomic subjects of neoplastic disease. The treatment methods are also shifted to pharmacogenomics and particular genetic and epigenetic profiles. Such perspectives are imperative to promote the outcomes of treatments in melanoma patients and escalate the efficacy of drugs against lethal cancers. To understand the dynamic transcriptional control of gene expression, it is crucial to gain knowledge about the functions of MITF and SOX10. Shifting to personalized treatment is in its beginning steps in melanoma treatment. Novel epigenetic medicines are expected to decline systemic toxicities by particular impacts. A comprehensive standpoint, which considers the phenotypes, genotypes, and epi-genotypes of melanoma cells, would be beneficial to understanding the sundry clinical behaviors and may aid us in developing novel therapeutic approaches. Despite the fact that epigenetic therapy for melanoma is still in its infancy, it is likely that their use will increase significantly in the future as single agents, combined with each other, or in combination with conventional chemotherapy. Therefore, we suggest that more investigations in the future will be valuable for examining the effects of the combination of epigenetic therapy with conventional and unconventional therapeutic approaches for the treatment of melanoma.

## Data Availability

All data and materials are within the paper.

## Abbreviations

NM: Nodular Melanoma
ALM: Acral Lentiginous melanoma
LM: Lentigo Maligna
SSM: Superficial Spreading Melanoma
MC1R: Melanocortin receptor
MEK: Mitogen-activated protein kinase
FGF: Fibroblast growth factor
NF-κB: Nuclear factor-κB
HIF-1: Hypoxia-Inducible Factor1
VEGF: Vascular endothelial growth factor
MAPK: Mitogen-activated protein kinase
RBD: RAS-binding domain
HDACs: Histone deacetylases
EZH2: Enhancer of zeste 2 Polycomb repressive complex 2 subunits
miRNAs: MicroRNAs
MMR: Mismatch repair
MGMT: O6-methyl-guanine-DNA methyltransferase
ABCB5: TP-binding cassette subfamily B member 5
CAF-1: Chromatin assembly factor 1
BAP1: BRCA1-associated protein-
PRMT1: Protein arginine methyltransferases 1
